# Editorial: Bradykinin and histamine mediated angioedema

**DOI:** 10.3389/falgy.2025.1732905

**Published:** 2025-11-14

**Authors:** Anant D. Patil, Jonny Peter, Michael Rudenko

**Affiliations:** 1 Department of Pharmacology, DY Patil Deemed to be University School of Medicine, Navi Mumbai, India; 2 Division of Allergy and Clinical Immunology, Department of Medicine, University of Cape Town, Cape Town, South Africa; 3 Allergy and Immunology Unit, University of Cape Town Lung Institute, Cape Town, South Africa; 4 London Allergy and Immunology Centre, London, United Kingdom

**Keywords:** hereditary, angioedema, urticaria, histamine, bradykinin

Angioedema is a condition characterised by localised, potentially life-threatening oedema of the subcutaneous and submucosal tissues. Although the clinical presentations of histamine- and bradykinin-mediated angioedema can be similar, their underlying pathophysiologies are distinct, necessitating precise diagnosis and fundamentally different treatment approaches. This is particularly critical in hereditary angioedemas (HAE), where delays in diagnosis can lead to severe complications, including fatal laryngeal oedema.

Spurred by recent advances in understanding these pathways and the development of novel therapies—from monoclonal antibodies to bradykinin receptor antagonists—this Research Topic was conceived to collate the latest evidence. We are pleased to present this collection of 11 articles from 60 international authors, featuring original research, reviews, and case reports that illuminate the evolving landscape of angioedema management.

## Synopsis of the research topic

The collection opens with a foundational review by Lima et al., which elegantly delineates the pathophysiology of both histamine- and bradykinin-mediated angioedema, providing essential context for the subsequent studies.

A significant focus of this issue is on refining diagnostics and understanding the real-world patient experience. Sexton et al. contribute a novel assay for quantifying high-molecular-weight kininogen (HKa), a promising step towards better biomarker development. Highlighting the challenges in clinical practice, Van der Poorten et al., in a nationwide Belgian study, report a median seven-year delay in diagnosing HAE, underscoring the need for greater awareness. Complementing this, Day et al. identify Black genetic ancestry and concomitant calcium channel blocker use as key risk factors for ACE inhibitor-induced angioedema and highlight the long median latency of treatment prior to first angioedema presentation (>5 years) in the majority of a South African cohort.

Several articles evaluate modern therapeutics. Bara et al. demonstrate the real-world efficacy of lanadelumab for long-term prophylaxis in Romanian patients, noting significant improvements in disease control and quality of life. Delving deeper into the mechanism of such treatments, Sexton et al., in a separate proteomic analysis, identify new potential biomarkers in patients undergoing lanadelumab therapy.

The importance of adaptable care models is addressed in two community case studies. Du et al. propose a standardised diagnostic and treatment workflow for resource-limited settings, while Andarawewa and Aygören-Pürsün emphasise the principle of individualised long-term therapy.

Finally, three compelling case reports illustrate these principles in action. Pinhal et al. successfully used subcutaneous C1-INH during pregnancy and lactation, and Guo et al. stress the value of family screening in identifying asymptomatic patients. Another report by Du et al. details the identification of a novel *SERPING1* gene mutation in a Chinese patient, leading to a definitive diagnosis and effective prophylactic treatment.

This Research Topic collectively underscores the rapid momentum in understanding bradykinin-mediated angioedema, while reaffirming the need for precise diagnosis and personalised care. We believe this compilation will serve as a valuable resource for clinicians, researchers, and students alike, ultimately contributing to improved outcomes for patients worldwide.

